# Burden of varicella complications in secondary care, England, 2004 to 2017

**DOI:** 10.2807/1560-7917.ES.2019.24.42.1900233

**Published:** 2019-10-17

**Authors:** James Lopez Bernal, Peter Hobbelen, Gayatri Amirthalingam

**Affiliations:** 1 Immunisation and Countermeasures Division, National Infection Service, Public Health England, Colindale, United Kingdom; 2 Department of Bacteriology and Epidemiology, Wageningen Bioveterinary Research, Lelystad, the Netherlands

**Keywords:** varicella zoster virus infection, chickenpox, vaccine, comorbidity, epidemiology, cost

## Abstract

**Background:**

Strategies to control varicella vary across Europe. Evidence from established programmes has prompted the United Kingdom to re-evaluate the need for universal vaccination. The burden of complicated varicella is a key parameter in the cost-effectiveness analysis.

**Aim:**

Our objective was to estimate the burden of complicated varicella in England.

**Methods:**

This electronic health record surveillance study used data from all NHS hospitals in England to identify varicella admissions between 2004 and 2017. The incidence of pre-defined complications of varicella was estimated using ICD-10 codes. Inpatient costs were calculated based on the payment rules for providers of NHS services.

**Results:**

There were 61,024 admissions with varicella between 2004 and 2017 and 38.1% had a recognised varicella complication. Incidence of hospitalisation increased by 25% and the proportion with complicated varicella by 24% from 2004/05 to 2016/17. The most common complications were bacterial skin infections (11.25%), pneumonia (4.82%), febrile convulsions (3.39%) and encephalitis (2.44%). Complication rates were higher in older age groups and the type of complications more severe. Length of stay for complicated varicella was 3.1 times longer than for uncomplicated varicella and inpatient costs were 72% greater.

**Conclusion:**

Complicated varicella has a substantial health and economic burden. These data together with data on impact on quality of life are important in informing the cost-effectiveness analysis of universal varicella vaccination.

## Introduction

Strategies to control varicella vary across Europe, with some countries including the United Kingdom (UK) adopting a selective programme and others (Finland, Germany, Greece, Italy, Latvia, Luxembourg and Spain) recommending universal vaccination using a two-dose schedule of the live-attenuated vaccine [1]. The United States and Canada also have longstanding two-dose schedules, whereas Australia has a single-dose schedule. The decision not to introduce a universal varicella vaccination programme in the UK has been primarily based on two factors: firstly, suggestions that vaccination of children may reduce a protective boosting effect in adults against herpes zoster; secondly, a reasoning that, while common, varicella infection in children generally causes mild, self-limiting illness, so the net benefits from the programme do not outweigh the potential harms from an increase in zoster [2,3]. Furthermore, there is a concern that, given the general perception of a mild disease, uptake of the vaccine may be suboptimal and could result in a shift of the age distribution of cases to older age groups where complication risks are higher.

Universal varicella vaccination is now being reconsidered in the UK for several reasons: firstly, more time has passed since varicella programmes were introduced in other countries, providing an opportunity to examine whether predicted increases in zoster have been seen. Secondly, the potential impact on zoster may be mitigated by the introduction of a zoster vaccination programme [4]. Thirdly, consideration of a one-dose schedule is more attractive and this had not previously been examined.

The UK’s national immunisation technical advisory group, the Joint Committee of Vaccines and Immunisations (JCVI), has identified the burden of complicated varicella as a key parameter that could impact on the decision as to whether vaccination is considered cost-effective [5]. Although it is true that in the majority of cases, varicella results in mild disease, it can nevertheless result in severe complications leading to hospitalisation and comorbidities.

These can range from dehydration and skin infections to pneumonia and meningitis [6]. Current data on the burden of such complications in the UK are limited. Further information on the burden of complicated varicella is particularly pertinent given that a single-dose schedule may reduce the severity of varicella without eliminating circulating varicella, therefore one would not expect an increase in rates of zoster hospitalisations [7].

The aim of this study was to estimate the burden of varicella complications seen in secondary care. We used routine hospital data on all hospitalisations in England to examine the burden of complicated varicella. We report data on the number and incidence of admissions, length of stay and costs of complicated varicella.

## Methods

This surveillance study used routine secondary care electronic health records.

### Hospital admissions

We extracted data on hospitalisations in National Health Service (NHS) hospitals in England from 1 April 2004 to 31 March 2017 from the Hospital Episodes Statistics (HES) database. Data in HES are recorded as finished consultant episodes; these are periods of inpatient care under one consultant. If a patient is transferred to another consultant or another hospital, this is considered a new episode. In order to estimate the number of admissions, we reclassified episodes into continuous inpatient spells so that if a patient was transferred to another consultant or hospital, this would not be considered a new admission. Varicella admissions were identified as those with an ICD-10 code for varicella (B01) in any diagnostic field of any episode within a continuous inpatient spell. We intentionally kept a broad definition of admissions associated with varicella because for many complicated cases of varicella, the primary reasons for admission are likely to relate to the complication yet varicella can be an underlying cause of each of the complications that we examine (discussed below). Admissions were categorised according to age, sex and method of admission (elective, emergency, maternity or other). Incidence was calculated using Office for National Statistics mid-year population estimates for England as a denominator [8]. Data are presented by financial year, which runs from April to March.

### Complications

The literature was reviewed in order to identify possible complications of varicella. We searched the Pubmed database for literature on varicella complications in hospitals. In order to focus on the most frequent complications, we searched for articles with the following combination of terms in the title field: hospital* AND (varicella OR chickenpox). This produced a list of 180 articles. We subsequently refined this selection by excluding (i) case reports, (ii) articles reporting on hospital complications after a national varicella vaccination programme had been implemented, (iii) articles that only identified varicella complications listed in the B01 section of ICD-10 and (iv) articles not written in English, French, Spanish, German or Dutch. From this refined selection, a total of 27 articles contained relevant information on varicella complications. A list of these complications was compiled and the associated ICD-10 codes were identified (Supplementary Table S1). Identification of these ICD-10 codes in any diagnostic field alongside a varicella diagnosis in any diagnostic field was considered a complication. Complications were then classified according to the following categories: cardiovascular, congenital, ear, nose and throat (ENT), fever, gastrointestinal, haematological, liver, musculoskeletal, neurological, ocular, renal, respiratory, sepsis, skin and other. Rates for each complication were calculated as a percentage of all varicella admissions.

### Immunodeficiency

We classified patients as immunocompromised according to ICD-10 codes as per Hobbelen et al. 2016 [4]. This included malignancies affecting the immune system, HIV, malignant neoplasms, transplantations, haematological and other conditions affecting the immune system. A full list of ICD-10 codes is provided in Supplementary Table S2.

### Inpatient costs

NHS inpatient spells are assigned a dominant Health Resource Group (HRG) for the episode. An HRG groups together patient events that are of similar cost. Inpatient costs were calculated according to the 2016/17 National Tariff [9]. Costs also take into account the type of admission (costs differ between elective or non-elective admission) and length of stay and are multiplied by a factor for local hospital provider market forces (an estimate of unavoidable cost differences based on geographical location) [9]. Before 2012, a different payment system was in place, therefore costs data were based on admissions from 1 April 2012. Costs do not include accident and emergency or critical care costs.

### Ethical approval

Public Health England has legal permission, provided by Regulation 3 of The Health Service (Control of Patient Information) Regulations 2002, to process patient confidential information for national surveillance of communicable diseases [10]. This includes PHE’s responsibility to monitor the safety and effectiveness of vaccines.

## Results

### Number and incidence of hospital admissions

Table 1 shows the number and incidence of admissions with varicella for each category of complication; Supplementary Table S3 contains the number and incidence for each individual complication. There were 61,024 hospital admissions with a coding for varicella between 1 April 2004 and 31 March 2017, a mean of 4,694 admissions per year (95% confidence interval (CI): 4,355–5,033). Of these, 38.14% had a known complication of varicella. There was an increasing trend in varicella hospitalisations from ca 4,000 cases per year in 2004/05 to ca 5,600 cases in 2016/17 (Supplementary Figure S1). This was not in line with population growth, with incidence of hospitalisation increasing by 25% from 80 per million per year in 2004/05 to 100 per million per year in 2016/17 (Figure). The proportion of varicella admissions with a recognised complication of varicella also increased from 34% in 2004/05 to 42% in 2016/17. There was a clear seasonal pattern of varicella with spring peaks (Supplementary Figure S2). The increase in varicella admissions was mostly driven by children. There was also an increase in those older than 80 years. In other adults, incidence decreased. All age groups except for the 60- to < 80-year-olds saw an increase in the percentage with complications. The biggest increases by complication category were skin complications, gastrointestinal complications, ENT complications and renal complications. Further details can be found in Supplementary Tables S4 and S5.

**Table 1:** Number and incidence of varicella hospitalisations, by complication category, England, 2004–2017 (n = 61,024)

	Number of admissions	Percentage among all varicella admissions	Number of admissions per year		Incidence per 1,000,000 per year	
			Mean	95% CI	Mean	95% CI
Category						
Cardiovascular	26	0.04	2.0	1.1–2.9	0.04	0.02–0.05
Congenital	49	0.08	3.8	1.8–5.7	0.07	0.03–0.11
Ear, nose and throat	1,728	2.83	132.9	102.9–162.9	2.50	1.98–3.02
Fever	1,426	2.34	109.7	91.0–128.4	2.07	1.75–2.39
Gastrointestinal	3,655	5.99	281.2	239.6–322.7	5.31	4.61–6.01
Haematological	961	1.57	73.9	60.1–87.7	1.39	1.16–1.63
Liver	91	0.15	7.0	5.3–8.7	0.13	0.10–0.16
Musculoskeletal	364	0.60	28.0	21.9–34.1	0.53	0.42–0.64
Neonatal	28	0.05	2.2	1.4–2.9	0.04	0.03–0.05
Neurological	5,388	8.83	414.5	389.3–439.6	7.86	7.46–8.27
Ocular	502	0.82	38.6	32.2–45.1	0.73	0.62–0.84
Other	345	0.57	26.5	21.8–31.3	0.50	0.42–0.59
Renal	1,795	2.94	138.1	113.0–163.1	2.60	2.18–3.03
Respiratory	3,324	5.45	255.7	241.6–269.7	4.85	4.61–5.10
Sepsis	1,279	2.10	98.4	75.4–121.4	1.85	1.45–2.25
Skin	6,864	11.25	528.0	422.2–633.8	9.94	8.13–11.76
Complications						
Any	23,274	38.14	1,790.3	1,582.2–1998.4	33.85	30.52–37.17
None	37,750	61.86	2,903.8	2,765.0–3042.7	55.09	53.20–56.98
**Total**	**61,024**	**100**	**4,694.2**	**4,354.6–5033.7**	**88.93**	**84.01–93.86**

CI: confidence interval.

The sum of complications in each category may be more than the total, as some cases had more than one complication.

**Figure:**
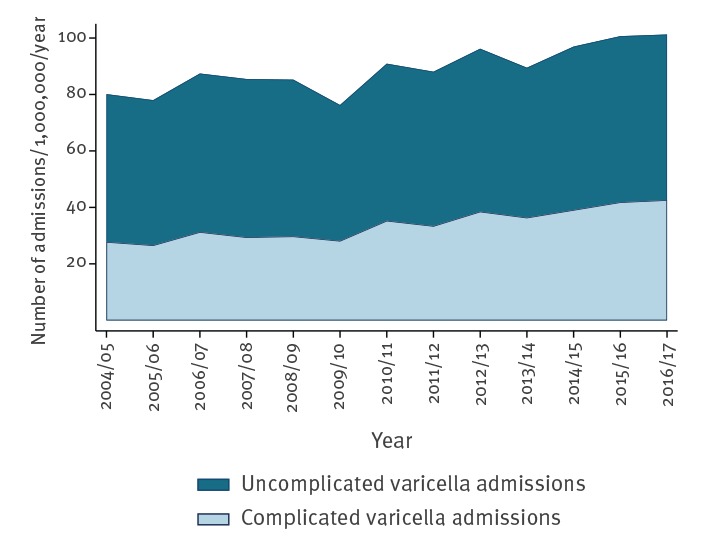
Annual incidence of varicella admissions, England, 2004–2017 (n = 61,024)

The most common category of complications were skin conditions (11.25% of admissions). Neurological complications were also common (8.83% of admissions), the largest groups of which were febrile convulsions (3.39% of admissions), encephalitis (2.44% of admissions) and meningitis (1.28% of admissions). Complications classified as gastrointestinal (5.99% of admissions) and respiratory complications (5.45% of admissions) were also common. Other common complications included fever (2.34% of admissions), tonsillitis (1.74% of admissions) and sepsis (1.49% of admissions). Cases of congenital and neonatal varicella were rare with just 0.08% and 0.05% of admissions, respectively.

### Age

Number and percentage of varicella admissions in each age group by category of complication are shown in Table 2. Incidence of varicella was highest in 0- to < 1-year-olds at 1,388 per million per year (95% CI: 1,290–1,486) and 1- to < 5-year-olds at 868 per million per year (95% CI: 818–918), dropped to 177 per million in 5- to < 10-year-olds (95% CI: 158–196) and remained between 10 and 30 per million beyond 10 years of age. Conversely, the proportion of incident admissions with complications was among the lowest in 0- to < 1-year-olds (30.64% of admissions) but highest in the older age groups (41.38% of admissions in 40- to < 60-year-olds; 54.91% of admissions in 60- to < 80-year-olds; 66.52% of admissions in > 80 year olds). The 1- to < 5-year-olds and 5- to < 10-year-olds also had higher rates of complications than infants (41.91% and 37.06% of admissions, respectively).

**Table 2:** Number and percentage of all varicella admissions in each age group, by complication category, England, 2004–2017 (n = 61,024)

Age group (years)	0–< 1		1–< 5		5–< 10		10–< 20		20–< 40		40–< 60		60–< 80		> 80		Unknown age	
	n	%	n	%	n	%	n	%	n	%	n	%	n	%	n	%	n	%
Category																		
Cardiovascular	3	0.03	7	0.02	7	0.10	1	0.04	4	0.07	3	0.13	1	0.06	0	0	0	0
Congenital	49	0.41	0	0	0	0	0	0	0	0	0	0	0	0	0	0	0	0
Ear, nose and throat	222	1.87	1,155	3.97	217	3.03	52	2.22	48	0.88	13	0.55	15	0.84	3	0.44	3	1.02
Fever	271	2.29	809	2.78	159	2.22	46	1.96	69	1.26	32	1.36	28	1.56	9	1.31	3	1.02
Gastrointestinal	905	7.63	1,702	5.86	382	5.34	144	6.15	191	3.49	100	4.25	123	6.86	90	13.10	18	6.14
Haematological	81	0.68	366	1.26	133	1.86	48	2.05	93	1.70	81	3.44	100	5.58	40	5.82	19	6.49
Liver	1	0.01	13	0.04	8	0.11	13	0.56	18	0.33	22	0.94	13	0.73	2	0.29	1	0.34
Musculoskeletal	34	0.29	206	0.71	55	0.77	9	0.38	7	0.13	22	0.94	17	0.95	11	1.60	3	1.02
Neonatal	28	0.24	0	0	0	0	0	0	0	0	0	0	0	0	0	0	0	0
Neurological	445	3.75	2,702	9.30	570	7.96	248	10.59	464	8.48	314	13.34	404	22.55	163	23.73	78	26.62
*Encephalitis	*28*	*0.24*	*423*	*1.46*	*264*	*3.69*	*75*	*3.20*	*128*	*2.34*	*101*	*4.29*	*308*	*17.19*	*115*	*16.74*	*46*	*15.70*
*Meningitis	*58*	*0.49*	*46*	*0.16*	*20*	*0.28*	*103*	*4.40*	*297*	*5.43*	*165*	*7.01*	*49*	*2.73*	*15*	*2.18*	*25*	*8.53*
Ocular	69	0.58	299	1.03	72	1.01	11	0.47	20	0.37	10	0.43	12	0.67	8	1.16	1	0.34
Other	33	0.28	230	0.79	44	0.62	6	0.26	4	0.07	6	0.26	14	0.78	6	0.87	2	0.68
Renal	109	0.92	497	1.71	304	4.25	98	4.18	154	2.81	151	6.42	256	14.29	205	29.84	21	7.17
Respiratory	470	3.96	952	3.28	247	3.45	120	5.12	569	10.40	416	17.67	338	18.86	183	26.64	29	9.90
*Pneumonia	*230*	*1.94*	*823*	*2.83*	*245*	*3.42*	*115*	*4.91*	*570*	*10.41*	*418*	*17.76*	*330*	*18.42*	*184*	*26.78*	*29*	*9.90*
Sepsis	243	2.05	563	1.94	134	1.87	42	1.79	67	1.22	84	3.57	109	6.08	28	4.08	9	3.07
Skin	1,186	10.00	4,453	15.32	779	10.88	110	4.70	84	1.54	94	3.99	86	4.80	56	8.15	16	5.46
Complications																		
Any	3,633	30.64	12,183	41.91	2,653	37.06	756	32.28	1,484	27.12	974	41.38	984	54.91	457	66.52	150	51.20
None	8,223	69.36	16,885	58.09	4,506	62.94	1,586	67.72	3,989	72.89	1,380	58.62	808	45.09	230	33.48	143	48.81
**Total**	**11,856**	**100**	**29,068**	**100**	**7,159**	**100**	**2,342**	**100**	**5,473**	**100**	**2,354**	**100**	**1,792**	**100**	**687**	**100**	**293**	**100**

The sum of complications in each category may be more than the total, as some cases had more than one complication.

In children, the proportion with ENT complications (1.87–3.97% of admissions), fever (2.22–2.78% of admissions) and skin complications (10.00–15.32% of admissions) was higher compared with older ages. A more detailed breakdown looking at individual complications showed that unspecified cellulitis and staphylococcal/streptococcal skin infections were among the most common individual complications in children up to 10 years of age (4.27–8.48% and 2.77–4.31% of admissions, respectively). Feeding problems were common in infants (n = 325, 2.74% of admissions) as was bronchiolitis (n = 209, 1.76% of admissions). Febrile convulsions and tonsillitis were particularly common in 1- to < 5-year-olds (n = 1,727, 5.94% of admissions and n = 764, 2.63% of admissions respectively). The 5- to < 10-year-olds were unusual in that they had high rates of nephrotic syndrome (n = 172, 2.40% of admissions) which was not seen in other age groups. They also had a high proportion of encephalitis compared with younger children, although, as a percentage of admissions, this was not as high as in older adults (> 40 years).

In adults and older children, the proportion with respiratory, neurological and renal complications was higher compared with younger children. In particular, in adults older than 40 years, these categories made up a very high proportion of the complications (respiratory: 17.67–26.64% of admissions; neurological: 13.34–23.73%; renal: 6.42–29.84%). Some of the more severe complications, including encephalitis, meningitis and pneumonia, made up the highest proportion of complications in adults and older children (Table 2). The proportion of varicella admissions complicated by encephalitis and pneumonia generally increased with age, with ca 16% of admissions complicated by encephalitis in those older than 60 years and pneumonia complicating 18–26% of admissions in those older than 40 years. Rates of urticaria, respiratory failure, acute renal failure and sepsis also generally increased with age and were common complications in adults. Meningitis tended to affect older children and younger adults, while it was a very rare complication in those younger than 10 years and rare in those older than 60 years.

Although many of these complications affected a greater percentage of varicella admissions in older ages, in terms of incidence and absolute numbers, the majority were higher in young children owing to the much larger number of admissions in these age groups. 

Further details of analysis by age group can be found in Supplementary Tables S6–S8.

### Sex

Number and percentage of varicella admissions for males and females by complication category is shown in Table 3. There were more varicella admissions among males compared with females (33,249 vs 27,773). There was little difference in the rates of complications among males and females for all complication categories. Regarding individual complications, Guillain–Barré syndrome was substantially more common in males (0.31% of admissions compared with 0.04% in females) as was viral croup (0.35% of admissions compared with 0.15% in females). Protein losing enteropathy (0.37% of admissions compared with 0.25% in females), nephrotic syndrome (0.85% if admissions compared with 0.60% in females), respiratory failure (0.73% of admissions compared with 0.53% in females) and bronchiolitis (0.43% of admissions compared with 0.31% in females) also appeared to be more common among males. Conversely, urticaria was the only complication that occurred frequently and appeared to be substantially more common among females (1.66% of admissions compared with 0.84% among males). Analysis by sex for individual complications can be found in Supplementary Table S9.

**Table 3:** Number and percentage of all varicella admissions, by sex and complication category, England, 2004–2017 (n = 61,022)

	Male		Female	
	n	%	n	%
Category				
Cardiovascular	14	0.04	12	0.04
Congenital	29	0.09	20	0.07
Ear, nose and throat	965	2.90	763	2.75
Fever	790	2.38	636	2.29
Gastrointestinal	1,981	5.96	1,674	6.03
Haematological	543	1.63	418	1.51
Liver	55	0.17	36	0.13
Musculoskeletal	227	0.68	137	0.49
Neonatal	12	0.04	16	0.06
Neurological	2,968	8.93	2,420	8.71
Ocular	291	0.88	211	0.76
Other	203	0.61	142	0.51
Renal	924	2.78	871	3.14
Respiratory	1,960	5.90	1,364	4.91
Sepsis	728	2.19	551	1.98
Skin	3,680	11.07	3,184	11.46
Complications				
Any	12,833	38.60	10,441	37.59
None	20,416	61.40	17,332	62.41
**Total**	**33,249**	**100**	**27,773**	**100**

Information on sex was missing for two cases.

The sum of complications in each category may be more than the total, as some cases had more than one complication.

### Immunosuppression

Number and percentage of varicella admissions by complication category for immunocompromised compared with immunocompetent patients is shown in Table 4. There were 4,457 varicella admissions in immunocompromised individuals (7.3% of admissions). Immunocompromised individuals were more likely to be admitted with uncomplicated varicella (69% compared with 61% of admissions in immunocompetent individuals). Immunocompromised patients with varicella had higher rates of fever, haematological complications, hepatic complications, renal complications, respiratory complications and sepsis. Conversely, they had lower rates of ear nose and throat complications, skin complications and neurological complications. Regarding individual complications, of those complications that occurred frequently (at least 0.5% of admissions), immunocompromised patients with varicella had higher rates of acute renal failure, sepsis, respiratory failure, pneumonia, encephalitis and urticaria but lower rates of staphylococcal or streptococcal skin infections, cutaneous abscesses, unspecified cellulitis and febrile convulsions. Analysis by individual complications can be found in Supplementary Table S10.

**Table 4:** Number and percentage of all varicella admissions by immunocompetence status and complication category, England, 2004–2017 (n = 61,024)

	Immunocompetent		Immunocompromised	
	n	%	n	%
Category				
Cardiovascular	23	0.04	3	0.07
Congenital	48	0.09	1	0.02
Ear, nose and throat	1,680	2.97	48	1.08
Fever	1,236	2.19	190	4.26
Gastrointestinal	3,417	6.04	238	5.34
Haematological	748	1.32	213	4.78
Liver	67	0.12	24	0.54
Musculoskeletal	341	0.60	23	0.52
Neonatal	28	0.05	0	0
Neurological	5,109	9.03	279	6.26
Ocular	470	0.83	32	0.72
Other	332	0.59	13	0.29
Renal	1,515	2.68	280	6.28
Respiratory	2,956	5.23	368	8.26
Sepsis	1,053	1.86	226	5.07
Skin	6,729	11.90	135	3.03
Complications				
Any	21,892	38.70	1,382	31.01
None	34,675	61.30	3,075	68.99
**Total**	**56,567**	**100**	**4,457**	**100**

The sum of complications in each category may be more than the total, as some cases had more than one complication.

### Admission method, length of stay and costs

More than 90% of admissions were emergency admissions, whether for uncomplicated varicella (91.8% of admissions) or complicated varicella (94.4% of admissions). Only for neonatal varicella were admissions most commonly from another source (babies born at the healthcare provider). Analysis by admission method is available in Supplementary Tables S11 and 12. Length of stay and cost by category is shown in Table 5. Varicella admissions with a complication had a mean length of stay of 6.6 days (95% CI: 6.2–6.9) and a mean inpatient cost of EUR 2,143 (95% CI: 2,068–2,219; GBP 1,893; 95% CI: 1,827–1,960) compared with 2.1 days (95% CI: 2.0–2.2) and EUR 1,272 (95% CI: 1,254–1,289; GBP 1,124; 95% CI: 1,108–1,139) for uncomplicated varicella. Costs were highest for neonatal varicella, cardiovascular and liver complications and lowest for congenital varicella, ENT complications and skin complications. Analysis by individual complication is available in Supplementary Table S13.

**Table 5:** Length of stay and inpatient costs for varicella admissions, by complication category, England, 2004–2017 (n = 61,024)

	Length of stay (days)		Cost (EUR)	
	Mean	95% CI	Mean	95% CI
Category				
Cardiovascular	28.8	18.6–39.0	9,697	6,420–12,974
Congenital	7.8	-1.4–17.0	998	890–1,107
Ear, nose and throat	2.6	1.8–3.5	1,576	1,138–2,014
Fever	5.8	4.8–6.9	2,280	1,994–2,566
Gastrointestinal	9.1	7.9–10.3	2,704	2,338–3,069
Haematological	19.3	16.4–22.2	4,407	3,817–4,997
Liver	36.1	25.4–46.8	8,133	4,245–12,020
Musculoskeletal	11.1	8.9–13.3	3,287	2,782–3,791
Neonatal	181.2	59.2–303.2	25,328	-4,152–54,808
Neurological	8.9	8.2–9.7	2,666	2,390–2,941
Ocular	5.4	2.5–8.2	2,220	1,027–3,413
Other	7.8	5.3–10.3	2,219	1,918–2,520
Renal	21.7	19.5–24.0	4,686	4,037–5,335
Respiratory	14.9	13.6–16.1	3,882	3,552–4,212
Sepsis	19.8	16.5–23.1	4,155	3,598–4,711
Skin	4.8	4.3–5.4	1,831	1,761–1,902
Complications				
Any	6.6	6.2–6.9	2,143	2,068–2,218
None	2.1	2.0–2.2	1,272	1,254–1,290
**Total**	**3.8**	**3.7**–**3.9**	**1,625**	**1,593**–**1,658**

CI: confidence interval.

## Discussion

We found that ca 38% of hospital admissions with varicella between 2004 and 2017 were associated with a recognised complication of varicella. Both the incidence of varicella hospitalisation and the proportion of admissions with complicated varicella increased over time. Among the most common complications were superadded bacterial skin infections, pneumonia, fever and febrile convulsions, encephalitis, nausea and vomiting, and dehydration. In general, complications were more common as a percentage of varicella admissions in older age groups and the type of complications tended to be more severe. Mean length of stay for varicella admissions with complications was more than three times longer than for uncomplicated varicella admissions and inpatient costs were around 70% greater.

Two previous studies that we are aware of have examined the incidence of varicella complications in the UK. Abdalrahman et al. also used HES data to examine trends in hospital admissions for varicella and zoster between 2001 and 2011, defining varicella admissions as those with varicella in the first two diagnostic fields [11]. The rates of complicated varicella were much lower (16–20%), however, that study only included three complications in their definition of complicated varicella (encephalitis, meningitis and pneumonia) as well as an undefined ‘other complications of varicella group’. Rates of encephalitis and pneumonia were similar to our estimates, although the rates of meningitis were lower. Cameron et al. used the British Paediatric Surveillance Unit active surveillance system to identify a defined list of severe varicella complications [12]. The study focussed on children under 16 years. Incidence of ataxia, necrotising fasciitis, purpura fulminans/disseminated coagulopathy, neonatal varicella and Reye’s syndrome was in line with the findings for 0- to 20-year-olds in our study (data on individual complications can be found in Supplementary Table S3). Nevertheless, rates of pneumonia, bacteraemia and encephalitis were substantially lower in the Cameron et al. study, while rates of toxic shock were slightly higher. This may reflect differences in case definitions, reporting methods, ascertainment or year-on-year variability (the Cameron et al. study focussed on a single year).

Elsewhere in Europe, rates of complications varied substantially. In Belgium, a study of paediatric inpatients with prospective case ascertainment and retrospective review of medical records in 2011/12 found higher rates of complications (65%) than our study, nevertheless, incidence of hospitalisation was lower. Similar to our study, they found lower rates of complications in infants compared with older children [13]. In the Netherlands, rates of complications were 34.9% among general practice (GP) patients in a study that searched free text and codes within GP electronic health records [14]. In Germany, a prospective active surveillance study with physician-completed questionnaires found complication rates of 79.6% among hospitalised children 16 years and younger, however, comparison with routine hospital data suggested that only 23% of varicella admissions were captured using the active surveillance method [6]. Dubos et al. also used prospective active surveillance in northern France and found a complication rate of 57% but incidence of complications was similar to that seen in our study [15]. Bonhoeffer et al. used active surveillance and patient records to identify varicella hospitalisations and found a complication rate of 74%, but found significant underascertainment of varicella cases [16]. Complication rates as a proportion of varicella cases were generally higher than average complication rates across years seen in our study, however, the highest complication rates tend to be seen when incidence is low, suggesting that there may be a higher threshold for admission in different health systems, or when ascertainment is low, suggesting that clinicians may be more likely to recall complicated cases than uncomplicated cases. In addition, differing case definitions also make comparisons across studies challenging.

The reason for the increase in hospitalisations and the increase in the percentage with complications over time is not clear. This could be due to improvements in coding over time in routine hospital data. The increase in hospitalisations could also be due to a shift in healthcare utilisation from primary to secondary care. This is supported by data showing a decrease in GP consultations for varicella between 2007 and 2014 [17]. We were unable to find studies with recent varicella trends from other countries that do not have a varicella vaccination programme. In countries that have introduced vaccination programmes, there has been a substantial decline in varicella [18].

Incidence of both complicated and uncomplicated varicella was highest in infants. This was not unexpected as they are least likely to have previously developed immunity through infection and tend to have a lower threshold for admission. We found that immunocompromised patients had a higher proportion of admissions without any recognised complications of varicella; the likely reason is a lower threshold of admission for immunocompromised patients with varicella, even if they do not have complications, as they are more likely to develop disseminated disease [19]. Another reason may be that these patients are more likely to be admitted for other reasons and varicella may have been incidental. Immunocompromised patients tended to have higher rates of some of the more severe complications.

It is likely that ascertainment of varicella admissions in our study was high: we included cases with varicella in any diagnosis code and completion of coding is generally high for HES data as it is used as the basis for provider payment and completion rates have been found to be over 85% for other diagnoses. Furthermore, ascertainment has been found to be high in international surveillance studies of varicella that used routine hospital data sources [6,16,20]. We included a broad range of varicella complications, based on existing evidence of a link to varicella. Moreover, we used a very large dataset covering all NHS admissions in England over 13 years. 

However, our study also has limitations. Our data relied on good coding of complications; complications which are less severe, such as fever or feeding problems, may be less likely to be coded than more severe complications such as septic shock or encephalitis. Importantly, we were unable to differentiate between those in whom varicella contributed to the complication and those in whom varicella was an incidental finding in an admission that occurred for a separate reason. Nor did we know the proportion of varicella that resulted in hospitalisation at different ages. For example, our finding that adults had higher rates of serious complications may be a result of the threshold for admission being higher for adults, so that those with uncomplicated varicella were less likely to be admitted. However, previous studies have shown that the probability of hospitalisation is much higher in those older than 10 years, therefore this is unlikely to be the case [4]. Finally, our cost estimates did not include costs for emergency department, critical care or out-of-hospital costs (for example primary care costs). 

We have not undertaken test statistics as the purpose of the study was to report on disease burden and costs. We had not made a priori hypotheses about the impact of other variables such as age, sex and immunosuppression. Testing each of these for each complication would require a large number of tests, many of which would be significant because of chance alone, therefore results would be difficult to interpret. As such we are wary of undertaking statistical tests which may be misleading.

Emerging evidence suggests that varicella vaccination has not been associated with an increase in herpes zoster cases [21,22]. It has been hypothesised that endogenous boosting from asymptomatic reactivation of varicella, following either natural infection or vaccination, may play an important role in the prevention of zoster [23]. Therefore, the effect of exogenous boosting may be less important than originally predicted.

Our study highlights that complicated varicella makes up a considerable proportion of varicella admissions in the UK and is associated with longer length of stay and higher costs. Moreover, the incidence of complicated varicella appears to be increasing. This presents a substantial health and economic burden. While incidence was highest in those younger than 1 year, 84% of complicated varicella cases were older than 1 year and thus may have been preventable by vaccination. Furthermore, over 94% of complicated varicella cases occurred in immunocompetent individuals eligible for live vaccine. Many of the immunocompromised cases may have been immunocompetent at the time of vaccination, and those not eligible for vaccination would be likely to benefit from herd immunity [24]. Findings from other countries suggest that severe complicated varicella could be prevented through either a one- or two-dose vaccination programme [7,25].

## Conclusion

The data presented here on the burden of complicated varicella in England should be used to inform analysis of the cost-effectiveness of a national varicella vaccination programme. This needs to be combined with data on the impact of complicated varicella on quality of life, which is currently being investigated. Rates of herpes zoster following the introduction of varicella vaccination programmes do not appear to have increased in most epidemiological studies, and herpes zoster vaccination programmes have now been established in the UK and elsewhere, which may protect some older adults. Our data show a substantial health and economic burden of severe complicated varicella and strengthen evidence to inform the introduction of a universal varicella vaccination programme [7,22].

## Supplementary Data

113000 SupplementaryTables

## Supplementary Data

100000 SupplementaryFigures
