# Supplementary material for: Burden of varicella complications in secondary care, England, 2004 to 2017
Source: Euro Surveill. 2019 Oct 17;24(42):1900233. doi: 10.2807/1560-7917.ES.2019.24.42.1900233 (PMC6807256; doi:10.2807/1560-7917.ES.2019.24.42.1900233)

Supplementary figures

Disclaimer: This supplementary material is hosted by Eurosurveillance as supporting information alongside the article "Burden of varicella complications in secondary care, England, 2004 to 2017" on behalf of the authors who remain responsible for the accuracy and appropriateness of the content. The same standards for ethics, copyright, attributions and permissions as for the article apply. Supplements are not edited by Eurosurveillance and the journal is not responsible for the maintenance of any links or email addresses provided therein.

Figure S1: Annual number of hospitalisations with uncomplicated and complicated varicella

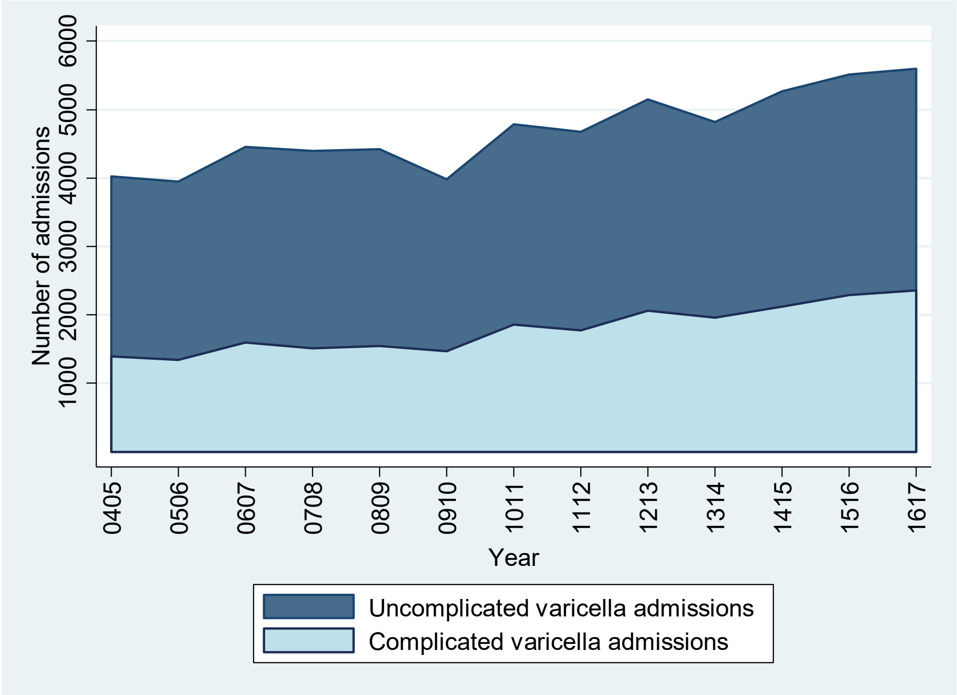

Figure S2: Annual number of hospitalisations with uncomplicated and complicated varicella

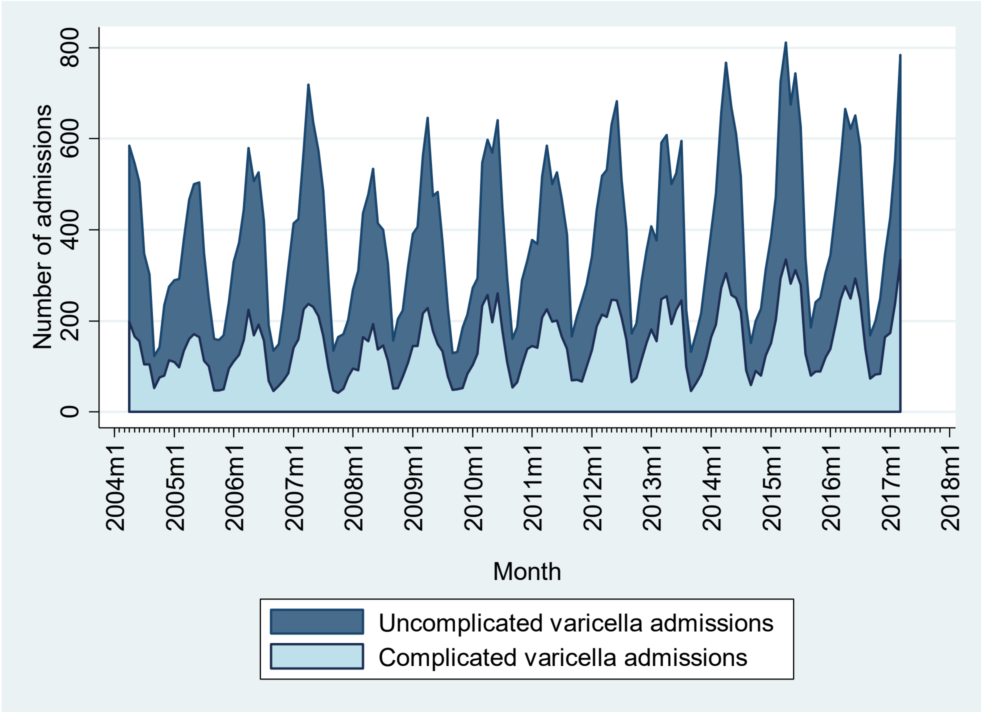

Supplement: SupplementaryFigures [file 19-00233_LOPEZ-BERNAL_SupplementaryFigures.pdf]
